# Fatal Human Case of Highly Pathogenic Avian Influenza A(H5N5) in a Backyard Flock Owner — Washington, November 2025

**DOI:** 10.15585/mmwr.mm7517a2

**Published:** 2026-05-07

**Authors:** Lynae Kibiger, Hanna N. Oltean, Lisa Leitz, Emma Krause, Debra Barrett, Anna Halloran, Kyle Yomogida, Beth Lipton, Keely Paris, Jared Keirn, Minden Buswell, Allison Black, Pauline Trinh, Theresa Murray, Roberto Bonaccorso, Leticia Banuelos, Ethan Dieringer, Jennifer Lenahan, Emily Spence Davizon, Ellyn P. Marder, Jocelyn Mullins, Meagan Kay, Eric J. Chow, Sandra J. Valenciano, John Lynch, Vanessa Makarewicz, Chloe Bryson-Cahn, Jennifer Hernandez, Kyla Haggith, Valicia Linn, Alex L. Greninger, Stephanie Goya, Sierra Gulla, Jennifer Young, Sierra Kerns-Funk, Brianna da Silva Bhatia, Hollianne Bruce, Krista Kniss, Katie Reinhart, Rachel Ohlstein, Shannon Johnson, Christina Schofield, Patrick Smith, Amber Itle, Maura Gibson, Brandi Torrevillas, Azeza Falghoush, Thomas B. Waltzek, Kevin Snekvik, Mia Torchetti, Timothy M. Uyeki, Scott Lindquist

**Affiliations:** ^1^Washington State Department of Health, Shoreline, Washington; ^2^Department of Epidemiology, University of Washington, Seattle, Washington; ^3^Grays Harbor County Public Health, Aberdeen, Washington; ^4^Public Health – Seattle & King County, Seattle, Washington; ^5^Division of Allergy and Infectious Diseases, Department of Medicine, University of Washington, Seattle, Washington; ^6^Division of Infectious Diseases, Department of Pediatrics, University of Washington, Seattle, Washington; ^7^Harborview Medical Center, Seattle, Washington; ^8^University of Washington Medical Center, Seattle, Washington;^ 9^Thurston County Public Health, Olympia, Washington; ^10^Clark County Public Health, Vancouver, Washington; ^11^Snohomish County Health Department, Everett, Washington; ^12^National Center for Immunization and Respiratory Diseases, Influenza Division, CDC; ^13^Harbor Regional Health, Aberdeen, Washington; ^14^MultiCare Capital Medical Center, Olympia, Washington; ^15^Washington State Department of Agriculture; ^16^Animal and Plant Health Inspection Service, Ames, Iowa; ^17^Washington Animal Disease Diagnostic Laboratory, Pullman, Washington.

**Summary**

**What is already known about this topic?**

Since 2022, highly pathogenic avian influenza (HPAI) A(H5) viruses have circulated among wild birds in the United States. Seventy human cases of influenza A(H5), most with mild illness, have been reported in the United States since 2024; 14 human influenza A(H5N1) cases were previously identified in Washington.

**What is added by this report?**

In November 2025, Washington reported the first human case of HPAI A(H5N5) infection worldwide. A positive laboratory result was obtained from a lower respiratory sample after multiple negative upper respiratory sample results; the patient experienced respiratory failure and died 28 days after symptom onset. The public health investigation identified approximately 135 exposed persons.

**What are the implications for public health practice?**

Symptom management and testing of exposed persons are critical to monitoring for human-to-human transmission of novel influenza infection. Environmental and animal investigations, including genomic analysis, can identify epidemiologic risk factors.

## Abstract

Clade 2.3.4.4b influenza A(H5N1) viruses have circulated across migratory bird flyways in the United States since 2022, including in Washington, where backyard flock detections have been reported annually. In November 2025, a Washington resident died from acute respiratory failure after receiving a positive influenza A(H5) test result at a hospital laboratory. Washington Public Health Laboratories confirmed influenza A(H5), and genomic sequencing identified influenza A(H5N5) virus (A6 genotype). Polymerase chain reaction testing detected highly pathogenic avian influenza A(H5) virus clade 2.3.4.4b from an apparently healthy backyard flock of ducks and sediment from a watering basin on the patient’s property. Six of eight gene segments from the environmental sample and one duck sample (partial neuraminidase segment) were highly genetically similar to the patient’s virus sequence. Although existing wild bird surveillance had not detected influenza A(H5N5) virus (A6) in the U.S. Pacific Flyway, introduction via wild birds into the environment of the backyard flock was likely the source of the patient’s exposure. The public health investigation identified approximately 135 exposed persons; symptom monitoring and influenza testing detected no additional cases. The overall risk for avian influenza A remains low among the general U.S. population; however, novel avian influenza A virus infection should be considered in persons with symptoms of influenza and potential exposures.

## Investigation and Results

### Illness Onset, Hospital Course, and Laboratory Testing

**Symptom onset.** In late October 2025, a Grays Harbor County, Washington, resident aged ≥65 years with a history of non-Hodgkin lymphoma developed fever, diarrhea, nausea, and cough (day 0) (Figure). The next day (day 1), the patient was evaluated in hospital A’s emergency department and discharged without a diagnosis. Nucleic acid testing (NAT) of a nares swab specimen was negative for influenza A virus, the first of multiple negative upper respiratory specimen influenza test results during the first 14 days of illness, which resulted in inconsistent implementation of isolation precautions.

**FIGURE:**
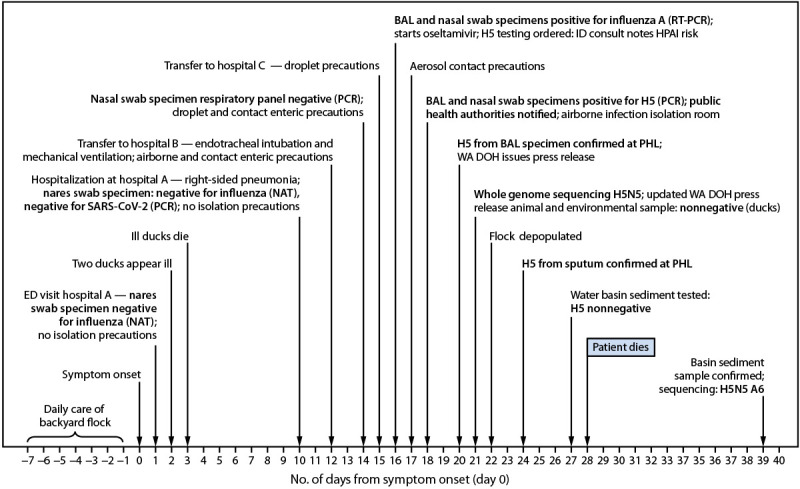
Timeline of events for fatal human case of avian influenza A(H5N5)* in a backyard flock owner — Washington, November 2025 **Abbreviations: **BAL = bronchoalveolar lavage; ED = emergency department; ID = infectious disease; HPAI = highly pathogenic avian influenza; NAT = nucleic acid test; PCR = polymerase chain reaction; PHL = public health laboratory; RT-PCR = reverse transcription–polymerase chain reaction; WA DOH = Washington Department of Health. * Nonnegative indicates a presumptive positive result, before confirmation by the National Veterinary Services Laboratory.

**First hospitalization (hospital A) and pneumonia.** On day 10, the patient was admitted to hospital A with confusion, an inability to walk, lower back pain, and a sore throat. A chest radiograph revealed right-sided pneumonia. A nares swab specimen tested negative for influenza by NAT.

**Clinical deterioration and intensive care (hospital B).** On day 12, because of worsening respiratory status, the patient was transferred to an intensive care unit (ICU) at hospital B and received endotracheal intubation, with invasive mechanical ventilation using a high efficiency particulate air (HEPA) filter. A nasal swab specimen tested with a respiratory viral polymerase chain reaction (PCR) panel was negative (day 14).

**First positive influenza A results (hospital C).** On day 15, the patient was transferred to hospital C’s ICU for an extracorporeal membrane oxygenation consultation. Bronchoalveolar lavage (BAL) and nasal swab specimens both tested positive for influenza A virus by reverse transcription–polymerase chain reaction (RT-PCR), and oseltamivir treatment was initiated (day 16). An infectious disease consult raised concern for avian influenza virus given the patient’s history of contact with a backyard flock. On day 17, aerosol contact precautions with eye protection were implemented (e.g., National Institute for Occupational Safety and Health–approved respirator, gown, gloves, and eye protection) (*1*,*2*).

**Identification of influenza A(H5N5), whole genome sequencing of BAL specimen, and death of patient.** On day 18, the University of Washington laboratory identified influenza A(H5) virus by PCR from the influenza A–positive BAL and nasal swab specimens. The patient was moved to an airborne infection isolation room, and the local health jurisdiction was notified. Washington Public Health Laboratories (WA PHL) confirmed the BAL influenza A(H5) subtyping result (collected on day 16) with a cycle threshold (Ct) value* of 25.26 (day 20). The University of Washington virology laboratory conducted whole genome sequencing of the BAL specimen and identified influenza A(H5N5) virus (genotype A6) (day 21). On day 24, WA PHL confirmed influenza A(H5) virus in a sputum sample (Ct = 28.00). Despite supportive critical care and aggressive treatment with influenza antiviral therapy (oseltamivir, baloxavir, amantadine, and peramivir), the patient died on day 28.

### Epidemiologic Investigation

**Illness in two ducks kept by patient.** The patient lived on a multiacre, rural property that was frequented by wild birds, including waterfowl. One family member lived in the house with the patient; another lived in a separate residence on the property. Grays Harbor County Public Health obtained exposure details via proxy interviews. The patient was the owner and primary caretaker of a backyard free-range poultry flock of 25 dabbling ducks^†^ and approximately 30 chickens, with multiple basins embedded in the ground for the birds’ enrichment and watering. During the week preceding symptom onset, the patient cared for the flock daily, handled eggs, and used a hose to clean and fill watering basins without using personal protective equipment (PPE). The patient owned no other animals, including livestock and had no known exposure to raw dairy products. On day 2 after the patient’s symptom onset, two ducks in the flock appeared ill; a household member removed them from the flock to a cage. These ducks died overnight and were disposed of on the property. The patient did not handle the ill or dead birds; daily care ceased at the time of the patient’s illness.

**Animal health investigation.** After notification by the Washington Department of Health (WA DOH), the Washington State Department of Agriculture (WSDA) and U.S. Department of Agriculture conducted an animal health investigation, including diagnostic sampling (day 21). Although the flock appeared healthy, oropharyngeal and cloacal swabs from all ducks on the property and a pooled sample of oropharyngeal swabs from chickens were collected and submitted to the Washington Animal Disease Diagnostic Laboratory (WADDL). The same day, WADDL reported weak Ct detections among all ducks by at least one of the following PCR assays: 1) avian influenza matrix PCR, 2) avian influenza A(H5) PCR, or 3) avian influenza (A[H5], 2.3.4.4) PCR; the pooled chicken swabs tested negative by avian influenza A(H5) PCR. The flock was depopulated on day 22. Positive specimens were forwarded to the National Veterinary Services Laboratories (NVSL), where six of the ducks tested positive for influenza A by PCR, all with Ct values >35; virus isolation and direct sequencing were unsuccessful.

**Environmental evaluation.** On day 21, WA DOH conducted an environmental investigation and collected samples at the patient’s property, including swabs from a feather, material suspected to be duck feces in the holding cage, and bottom sediment from one watering basin. The sediment tested nonnegative (presumptive positive result before confirmation)^§^ for avian influenza A(H5) clade 2.3.4.4b by PCR at WADDL (day 27) and was forwarded to NVSL, which confirmed highly pathogenic avian influenza (HPAI) A(H5), isolated the virus, and characterized the virus as A(H5N5), genotype A6 (partial genome with six of eight genes sequenced) (day 39).

A partial genome from one duck sample (Ct = 35.07; partial neuraminidase segment) was obtained by WADDL. The viral sequences from the sediment sample and the duck were highly genetically similar to the patient’s viral genome sequence.

## Public Health Response

A tiered health care personnel (HCP) risk assessment was generated in coordination with CDC, WA DOH, and local health jurisdictions as an option for health care partners in the event of staffing shortage concerns (Box) (*3*). HCP (124), family contacts (seven known), and state and federal government employees (four) with possible exposure to the patient, flock, or flock’s environment were monitored for influenza symptoms through day 10 after their last exposure. During symptom monitoring, 15 exposed persons developed compatible symptoms; none received positive test results for influenza (Table). Monitoring was primarily conducted using Research Electronic Data Capture (REDCap; version 16.0.18) databases with automated daily text message questionnaires.

BOX Risk assessment and recommendations* for health care personnel in facilities that cared for a patient with a fatal case of avian influenza A(H5N5) infection,^†^ by risk level of exposure — Washington, November 2025

**High-risk exposure: exposure to aerosols or aerosol-generating procedures without respirator or eye protection, or open ventilator circuit or no high-efficiency particulate air filter without respirator or eye protection**

Recommendations:

- Furlough through 10 days after last exposure, or
- May continue working under the following conditions:
  - Influenza molecular assay result for upper respiratory tract specimen is negative, and
  - Postexposure antiviral chemoprophylaxis started within 2 days of exposure, and continued through 10 days after last exposure
  - Face mask for source control and
  - Continued daily active symptom monitoring

**Moderate-risk exposure: exposure using eye protection and surgical mask (no respirator or non–fit-tested respirator)**

Recommendations:

- May continue working under the following conditions through 10 days after last exposure:
  - Face mask for source control and
  - Continued daily active symptom monitoring

**Low-risk exposure: exposure while using a fit-tested respirator and eye protection**

Recommendations:

- May continue working under the following conditions through 10 days after last exposure:
  - Passive symptom self-monitoring and
  - Face mask not required

* These recommendations from the state of Washington were developed during this public health investigation in collaboration with the health care facility, adapted from CDC guidance. Interim guidance for infection control within healthcare settings | Avian influenza (bird flu) | CDC

^†^ Being within 6 ft (1.8 m) of the patient or in the patient’s room for ≥15 minutes, regardless of whether personal protective equipment was used.

**TABLE:** Symptom monitoring and influenza test results among health care personnel, family contacts, and government employees exposed to a patient with a fatal case of highly pathogenic avian influenza A(H5N5) — Washington, November 2025

Characteristic	Health care personnel* (124)	Family contacts^†^ (7)	Government employees^§^ (4)	Total (135)
**Total monitored for symptoms**	**124**	**6**	**4**	**134**
Accepted postexposure prophylaxis	0	2^¶^	0	**2**
Developed respiratory symptoms	19	1	0	**20**
Negative influenza test result**	14	1	NA	**15**
Positive influenza test result**	0	0	NA	**0**
Not tested for influenza	5	0	NA	**5**

**Abbreviation:** NA = not applicable.

* High-risk exposure (exposure to aerosols or aerosol-generating procedures without respirator or eye protection, or open ventilator circuit or no high-efficiency particulate air filter without respirator or eye protection) or moderate-risk exposure (exposure using eye protection and surgical mask [no respirator or non–fit-tested respirator]).

^†^ One person lived with the patient in the same household, and a second person lived in a separate residence on the property; additional family members visited the patient in the hospital.

^§^ Public health and agricultural employees.

^¶^ Asymptomatic family contacts who lived on the property.

** Nasopharyngeal swab specimen.

Public health officials identified seven family members with exposure to the patient or the property, including two living on the property grounds. Six family members agreed to passive weekly monitoring by phone. During the patient’s final hospitalization, only family visitation was permitted; the hospital provided PPE, including non–fit-tested respirators. Additional family members were identified through hospital visitation logs but contact information was not provided. Thus, the total number of exposed family members is unknown.

## Discussion

This detection of influenza A(H5N5) in a Washington resident is the first human influenza A(H5N5) virus detection worldwide. Diagnosis of influenza A(H5N5) clade 2.3.4.4b, genotype A6, in this patient resulted in a multijurisdictional public health response to ascertain exposure sources, identify exposed persons, and monitor for additional cases. WA DOH and WSDA sampling identified influenza A(H5N5) virus in the backyard flock environment and in apparently healthy ducks on the patient’s property. Although existing federal and state-based wildlife surveillance had not detected influenza A(H5N5) virus (genotype A6) in the U.S. Pacific Flyway, introduction of the virus into the environment of the backyard flock via wild waterfowl and the presence of amplifying hosts on the property were the most likely sources of exposure. The overall avian influenza risk to the general U.S. population remains low (*3*).

Avian influenza A viruses pose a higher human transmission risk when direct or close and prolonged exposure to infected poultry or other infected animals occurs without recommended PPE use (*2*). However, three cases of human infection with HPAI A(H5N1) viruses without a clear exposure source have been identified in the United States (*4*). To assist in early identification, appropriate treatment, and isolation, HCP should routinely inquire about relevant exposures, including contact with ill or dead animals or their environments, consumption or handling of raw animal products, and contact with a confirmed or suspected human case of avian influenza virus infection when evaluating patients with acute respiratory illness (particularly those with severe illness requiring hospitalization) (*2*,*5*).

The diagnosis of influenza A(H5N5) virus infection in the patient described in this report was complicated by early and repeated negative influenza test results from upper respiratory swab specimens. Negative influenza results from initial upper respiratory specimens have been described in three similar patients with lower respiratory tract disease hospitalized with avian influenza A(H5N1) infection (*4*). Thus, avian influenza virus infection should not be ruled out in hospitalized patients based on negative influenza laboratory test results from upper respiratory tract specimens if the patients have lower respiratory tract disease, relevant exposures, and no confirmed etiology for their disease. If avian influenza virus infection is suspected in a patient with severe respiratory disease, both upper and lower respiratory tract specimens should be collected for influenza testing by RT-PCR at a public health laboratory (*6*).

Early negative influenza results delayed initiation of isolation precautions, reporting to public health authorities, and symptom monitoring. Although isolation precautions were not established consistently until the ninth day of inpatient care, no cases among HCP were identified. Likewise, no cases were detected among family members, despite lengthy exposure to both the symptomatic patient and the property. One household member reported direct contact with the ill and dead ducks but remained asymptomatic. Establishing a tiered risk assessment for HCP exposures based on setting and PPE use allowed staff members to continue working while having their symptoms monitored and limited new HCP exposures. The investigation was complicated by its occurrence during viral respiratory season and symptom development among several persons whose symptoms were being monitored. Human-to-human transmission of avian influenza A viruses has only rarely been reported globally and has not been reported in the United States (*3*,*7*).

Timely HPAI risk evaluation is important for persons with influenza symptoms requiring hospitalization to support infection prevention and control, early notification of public health authorities, and robust epidemiologic investigation, including genomic sequencing to identify possible transmission pathways. Ill or dead animals should be reported to animal health authorities for surveillance and potential testing and to reduce human exposure. Public health guidance for evaluating suspected cases of avian influenza should include immediate isolation precautions, prompt initiation of antiviral treatment, repeated influenza testing, and specimen collection from multiple sites (*2*,*6**,**8*). Considering the successive influenza A–negative laboratory results in the Washington patient, sampling from both upper and lower respiratory tracts in hospitalized patients should be considered to increase the likelihood of laboratory detection.

## Footnotes

* The number of PCR cycles necessary to detect a virus, with lower Ct values indicating a higher concentration.

† Surface-feeding waterfowl that tip forward to graze on shallow plants, insects, and larvae.

§ Nonnegative indicates a presumptive positive result, before confirmation by the National Veterinary Services Laboratory.
